# Supplementary material for: Metabolomic Study of a Rat Model of Retinal Detachment
Source: Metabolites. 2022 Nov 7;12(11):1077. doi: 10.3390/metabo12111077 (PMC9699637; doi:10.3390/metabo12111077)
Supplement: Supplementary file 1 [file metabolites-12-01077-s001.zip › Supplemenraty Table S1 Abbreviations.pdf]

Table S1. Summaries of the Abbreviations and Full names in the text

Abbreviations	Full Name
RD	Retinal detachment
GEO	Gene Expression Omnibus
ACLY	ATP-citrate lyase enzyme
RPE	retinal pigment epithelium
PVR	proliferative vitreoretinopathy
AGC	automatic gain control
IT	injection time
KEGG	Kyoto Encyclopedia of Genes and Genomes
PLS-DA	partial least squares discriminant analysis
OPLS-DA	orthogonal partial least squares discriminant analysis
LOOCV	leave-one-out cross-validation
VIP	variable importance
PCA	Principal component analysis
FC	fold change
SAH	S-adenosylhomocysteine
SAM	S-adenosylmethionine
XDH	xanthine dehydrogenase
PFK1	phosphofructokinase 1
PDH1	pyruvate dehydrogenase 1
PHA	phenylalanine hydroxylase
TH	Tyrosine hydroxylase
L-DOPA	L-3,4-dihydroxyphenylalanine
BRB	blood–retinal barrier
ICMT	isoprenylcysteine carboxyl methyltransferase
XO	Xanthine oxidase
PNP	purine nucleoside phosphorylase
IMPDH1	inosine-5′-monophosphate dehydrogenase 1
IMP	inosine monophosphate
TYR1	tyrosinase-related protein1
PDH	pyruvate dehydrogenase
TCA	tricarboxylic acid
