# Supplementary figures and images for: Metabolomic Study of a Rat Model of Retinal Detachment
Source: Metabolites. 2022 Nov 7;12(11):1077. doi: 10.3390/metabo12111077 (PMC9699637; doi:10.3390/metabo12111077)

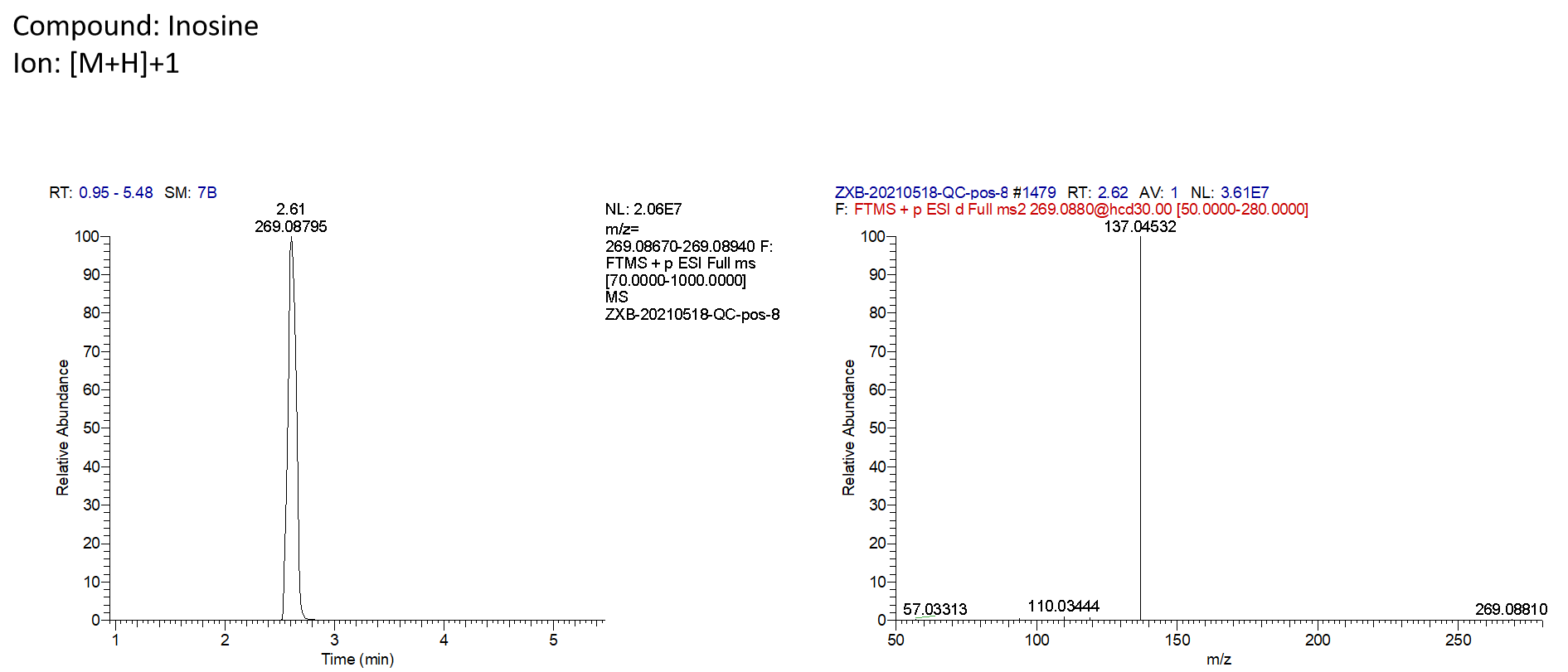

Supplement: Supplementary file 1 [file metabolites-12-01077-s001.zip › Supplemenraty Figure S1/SF1-AInosine.png]

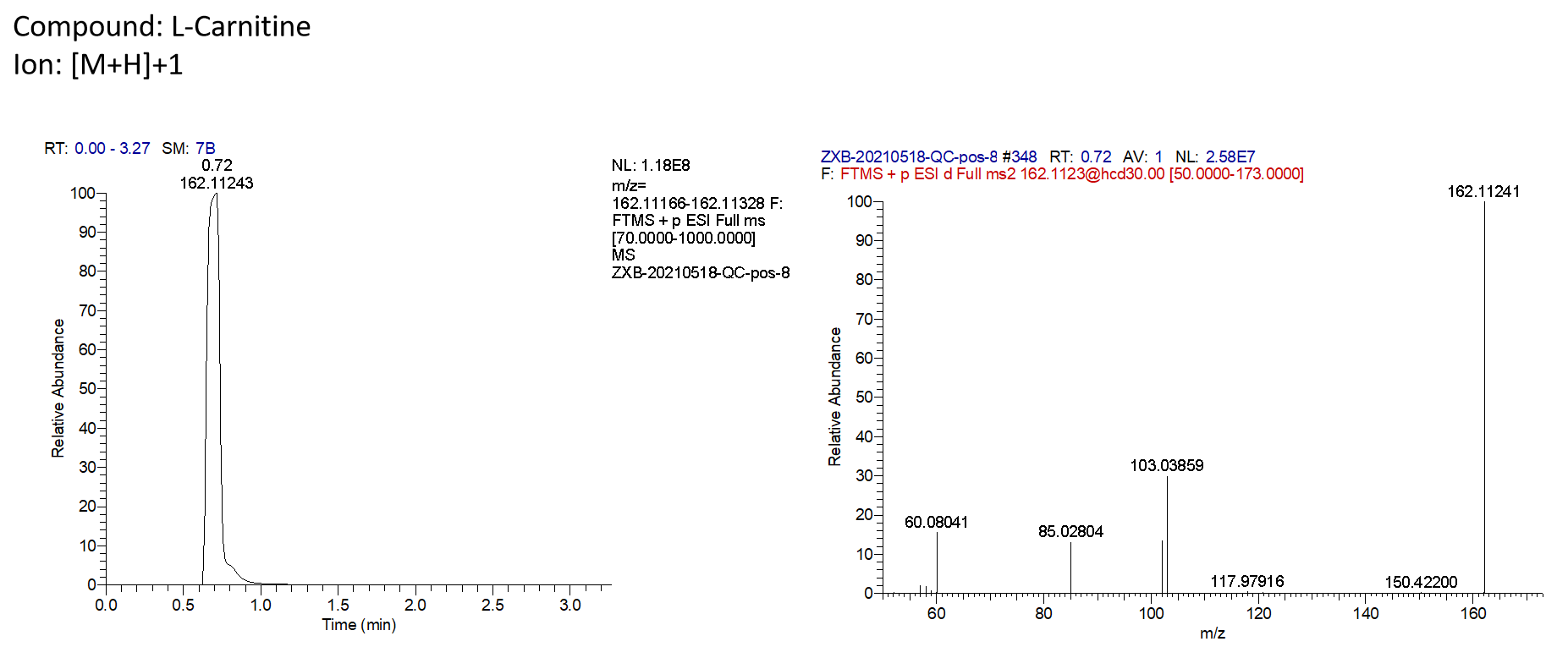

Supplement: Supplementary file 1 [file metabolites-12-01077-s001.zip › Supplemenraty Figure S1/SF1-BL-Carnitine.png]

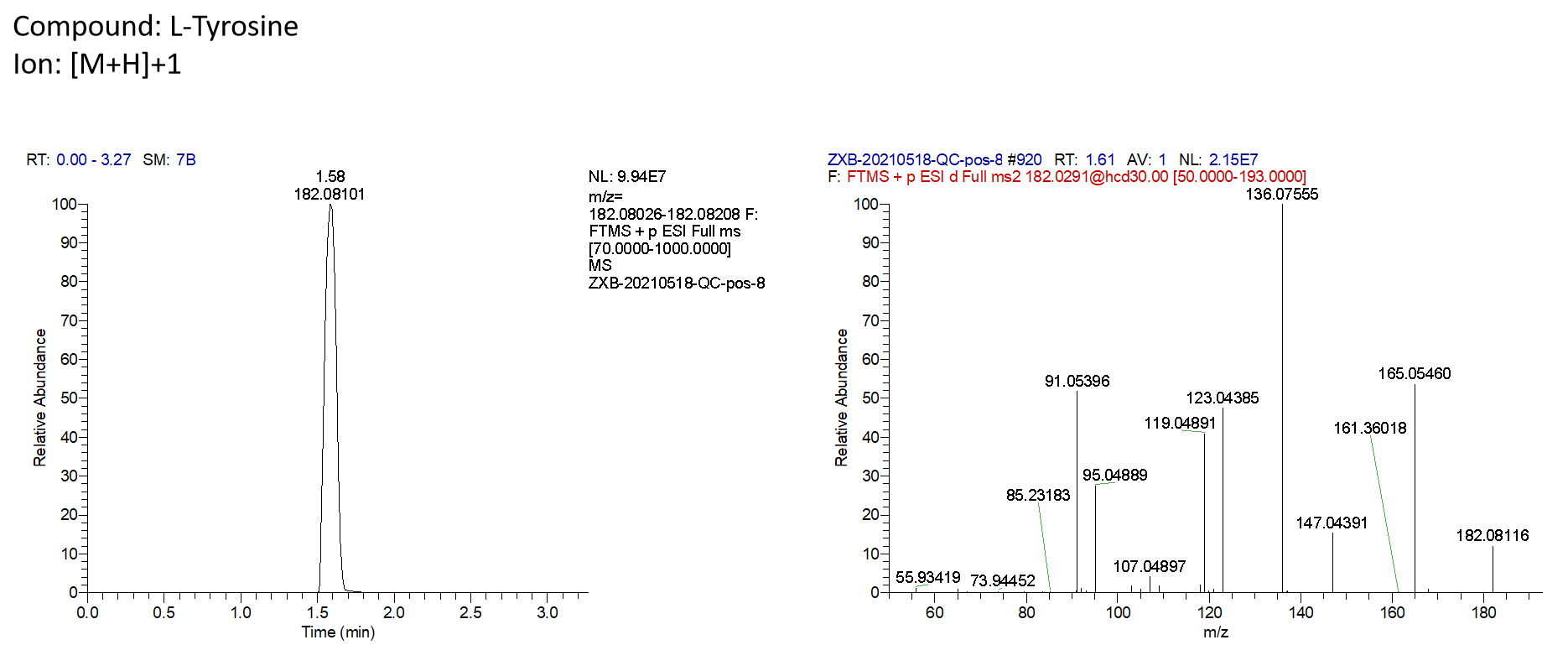

Supplement: Supplementary file 1 [file metabolites-12-01077-s001.zip › Supplemenraty Figure S1/SF1-CL-Tyrosine.png]

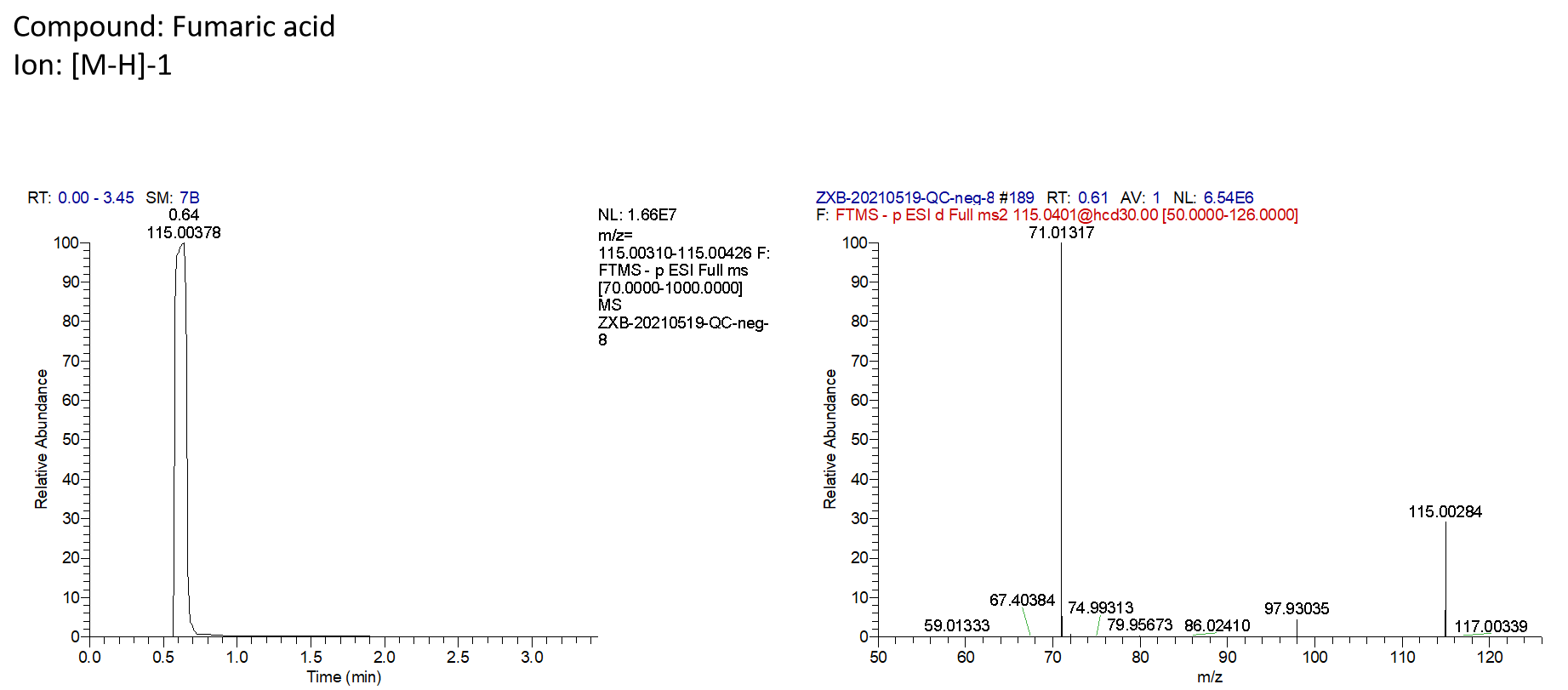

Supplement: Supplementary file 1 [file metabolites-12-01077-s001.zip › Supplemenraty Figure S1/SF1-DFumaric acid.png]

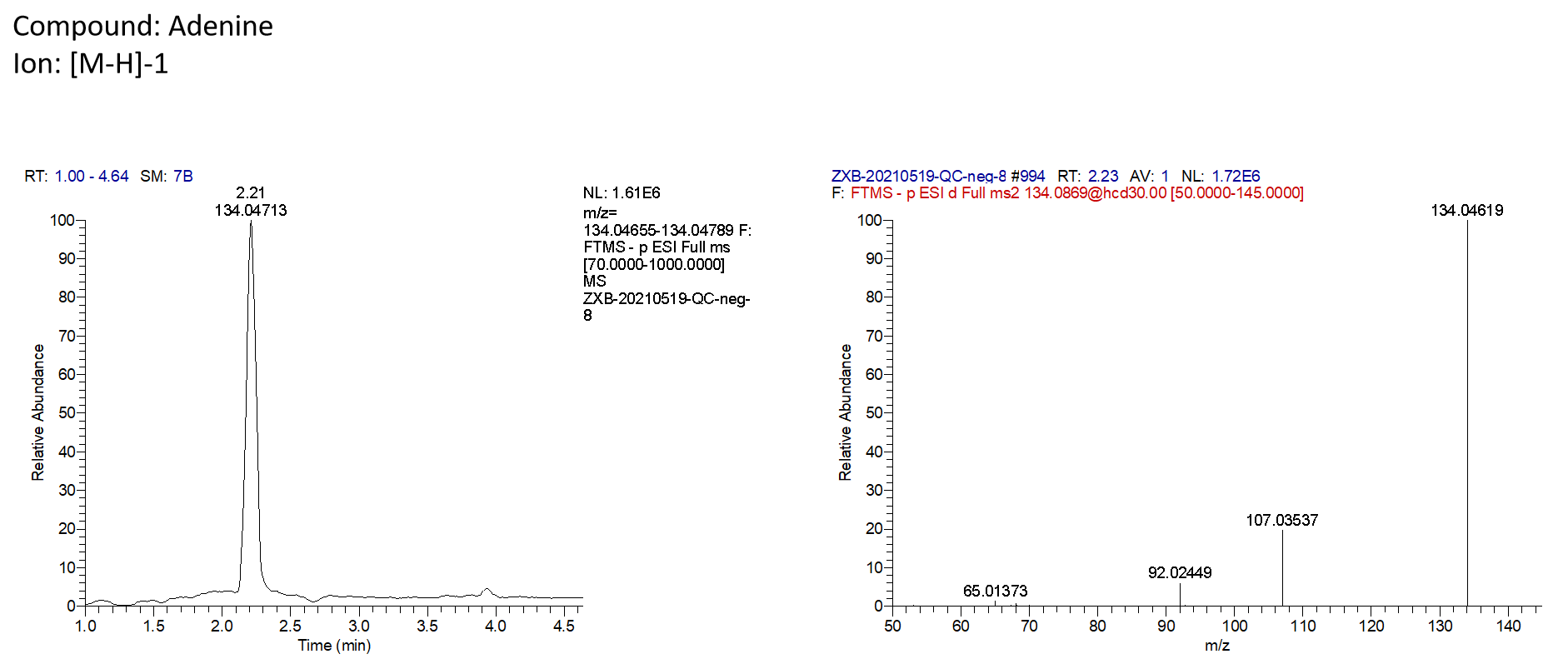

Supplement: Supplementary file 1 [file metabolites-12-01077-s001.zip › Supplemenraty Figure S1/SF1-E Adenine.png]

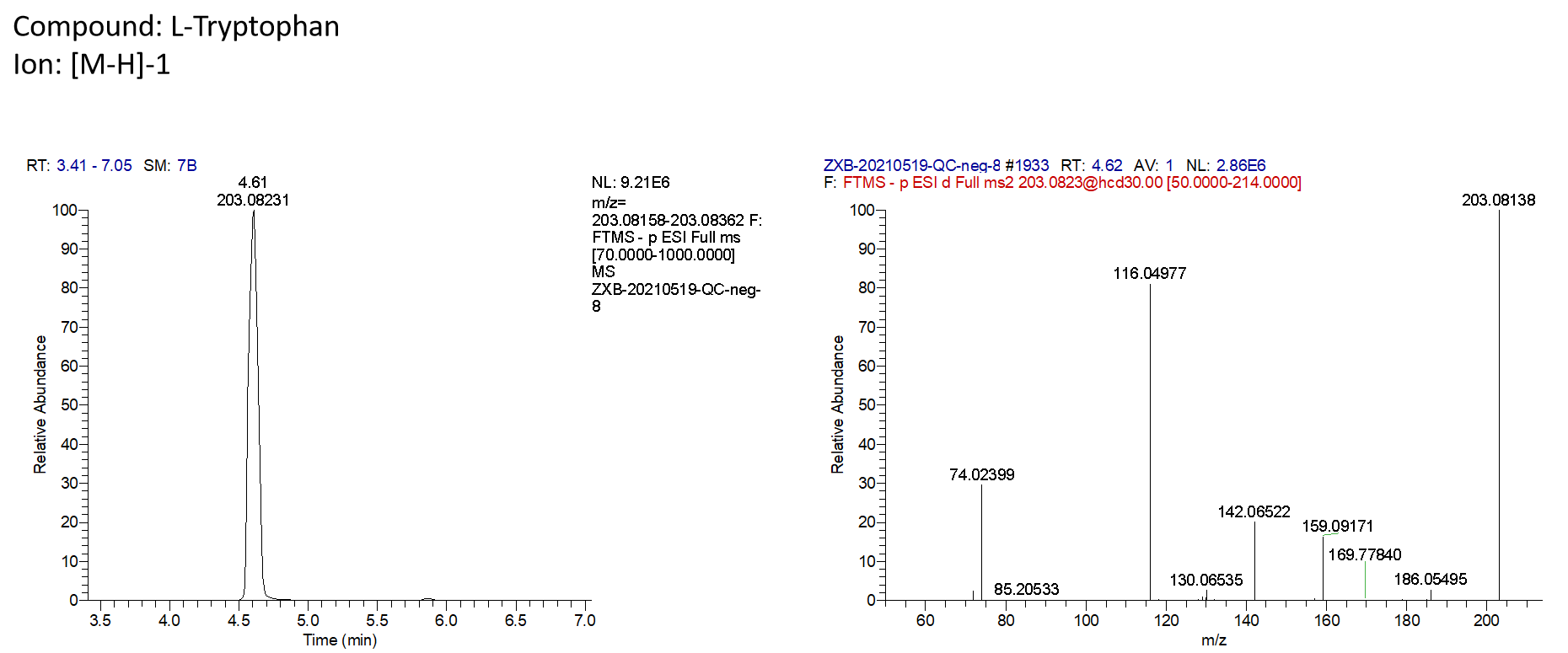

Supplement: Supplementary file 1 [file metabolites-12-01077-s001.zip › Supplemenraty Figure S1/SF1-F L-Tryptophan.png]

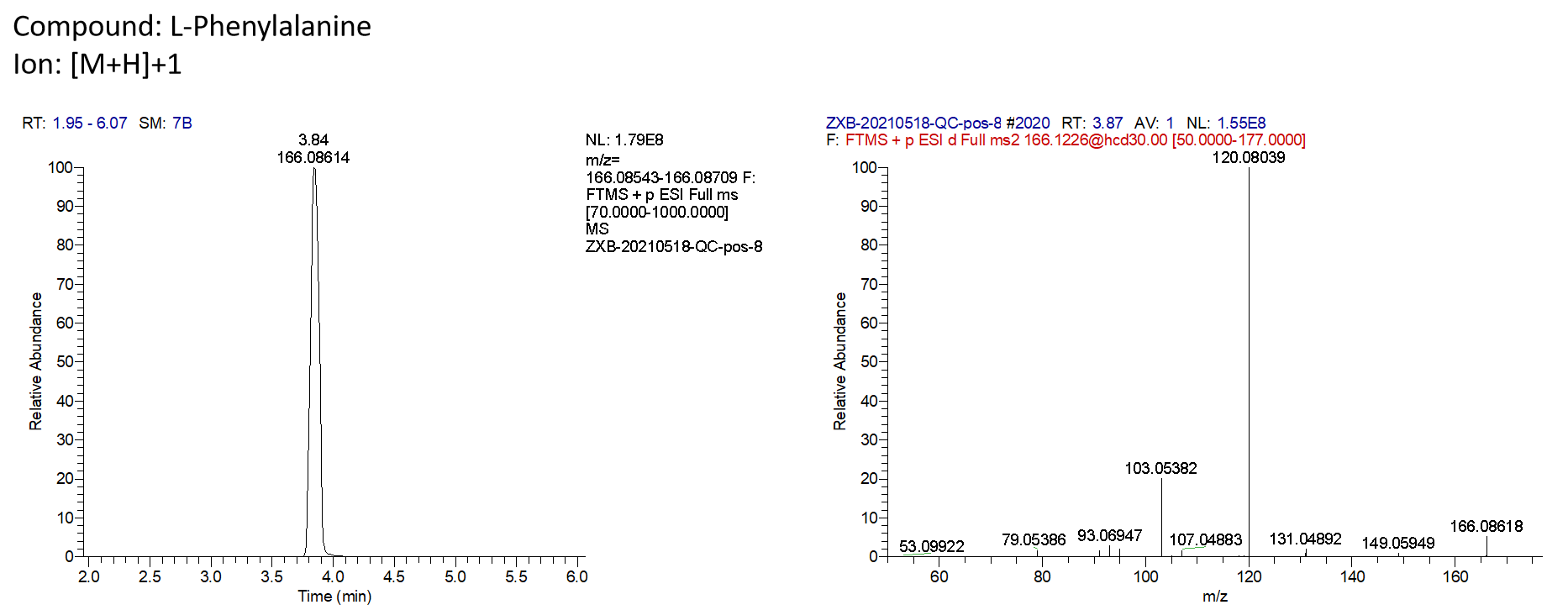

Supplement: Supplementary file 1 [file metabolites-12-01077-s001.zip › Supplemenraty Figure S1/SF1-G-Phenylalannine.png]

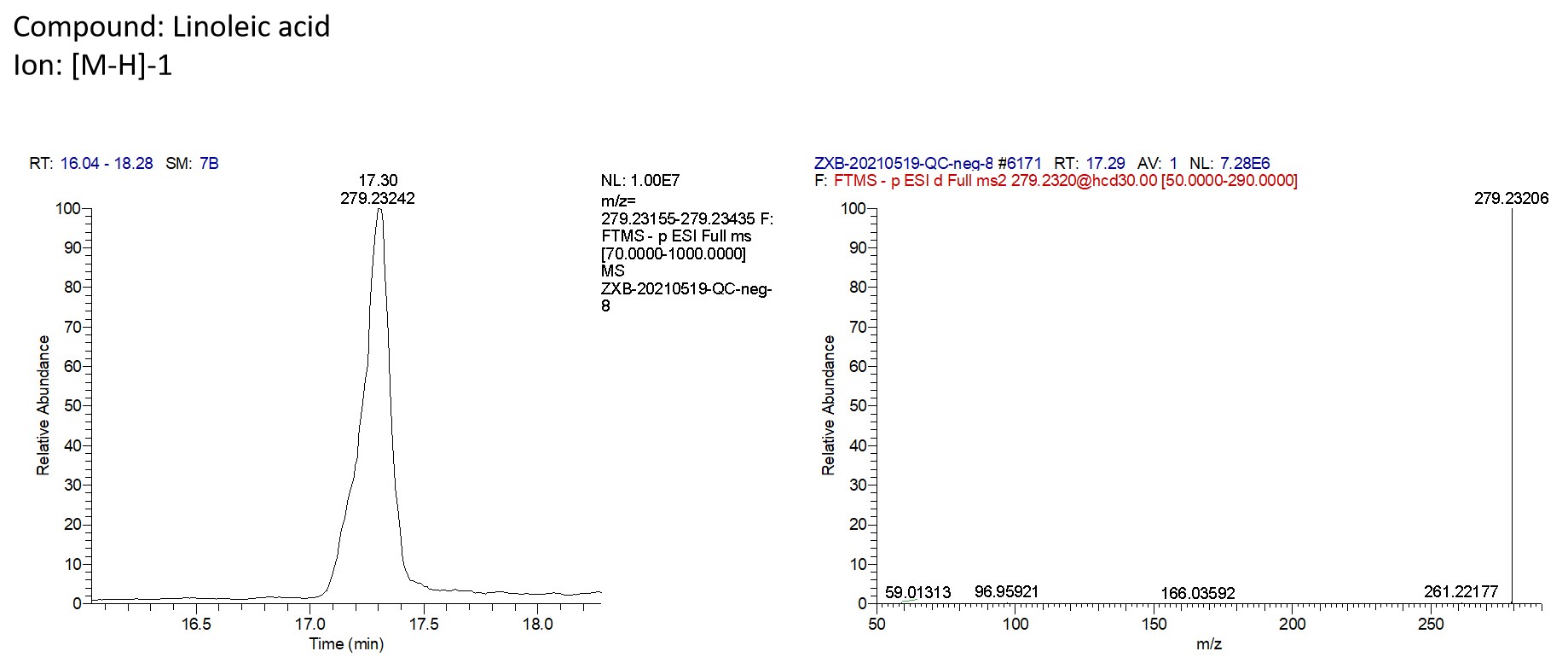

Supplement: Supplementary file 1 [file metabolites-12-01077-s001.zip › Supplemenraty Figure S1/SF1-H Linoleic acid.png]
